# Smile Beyond the Stars: A Narrative Review Exploring the Challenges for Dentistry in Space

**DOI:** 10.7759/cureus.66591

**Published:** 2024-08-10

**Authors:** Jumana Hasin Abdul Samathu, Rekha Mani, Vijay Venkatesh, Abinia Vaishnavi, Lavanya Sacrapani

**Affiliations:** 1 Dental Surgery, SRM Kattankulathur Dental College and Hospital, Chennai, IND; 2 Conservative Dentistry and Endodontics, SRM Kattankulathur Dental College and Hospital, Chennai, IND; 3 Conservative Dentistry and Endodontics, Faculty of Dental Science, King George's Medical University, Lucknow, IND

**Keywords:** dental procedures, dentistry, oral health, microgravity, aerospace

## Abstract

Space dentistry addresses the unique challenges of providing dental care in space, where zero gravity, limited resources, and the vast distance from Earth complicate the maintenance of oral health. Ensuring astronauts' dental health is crucial, as dental issues can adversely affect their overall health and mission performance. Microgravity exacerbates risks for dental problems such as periodontitis, dental caries, bone loss, and potentially, neoplasms. Traditional dental care methods become less effective in microgravity, leading to increased plaque accumulation and worsening of dental diseases.

As space missions venture further and last longer, maintaining oral hygiene presents unique challenges that necessitate innovative solutions. These include specialized tools like ergonomic toothbrushes and 3D-printed dental prostheses designed to function effectively in a zero-gravity environment. Preventive measures, such as comprehensive astronaut training programs focusing on oral health, are vital. These programs educate astronauts on maintaining oral hygiene and managing potential dental issues using available resources.

Collaborative efforts among dental professionals, engineers, and space agencies are essential to developing comprehensive strategies for space dentistry. Such interdisciplinary collaboration leads to the advancement of dental care technologies and methodologies that can address the unique needs of astronauts. Despite the formidable challenges, these innovative solutions and collaborative efforts offer promising avenues for ensuring the dental health of astronauts during long-duration missions. This review aims to examine the detrimental effects of microgravity on the oral cavity and explore potential solutions to these issues, ensuring that humanity can continue to push the boundaries of space exploration while safeguarding the well-being of those who venture into the cosmos.

## Introduction and background

Space exploration introduces unique health challenges, notably in dentistry, due to the extreme conditions beyond Earth’s atmosphere. Microgravity notably affects the human body, altering fluid distribution, musculoskeletal structure, and the functioning of vestibular and sensorimotor systems, necessitating special medical considerations. These physiological changes are compounded by altered microbial flora and immunodeficiency, significantly affecting dental health [1-2].

In space, common terrestrial dental issues such as caries, periodontitis, and bone loss present great risks due to the microgravity environment, which complicates their prevention and treatment [3]. Events from previous space missions reveal that dental problems have led to significant discomfort and have sometimes compromised mission objectives [4-7]. This underscores the lack of preparedness for dental emergencies in earlier missions, with anecdotal evidence of cosmonauts suffering from severe dental pain and the need for improved dental care in space [5].

As human endeavors in space continue to expand to longer and more distant missions, understanding and addressing dental care in microgravity becomes crucial. This review delves into the detrimental effects of space conditions on oral health, discusses the adaptations necessary for dental care in such environments, and explores the solutions to ensure the well-being of astronauts on long-term missions. This exploration into space dentistry highlights the specific challenges faced and underscores the interdisciplinary efforts required to tackle them, from advanced medical systems to innovative dental treatment methods [8].

## Review

Methodology

A comprehensive search was undertaken using MEDLINE, PubMed, and Google Scholar to conduct this review. Keywords employed included "aerospace," "microgravity," "space flight," "space mission," "zero gravity," "oral health," "dentistry," "preventive measures," and "space-adapted dental technologies." Owing to the limited availability of research work, related to the subject of interest, all existing literature, between 1983 and 2023 was included in the review. The search results were supplemented by reviewing the bibliographies of the articles found, ensuring a thorough exploration of the subject matter related to dental care challenges and innovations in the context of space missions.

Oral health and challenges in microgravity

**Figure 1:**
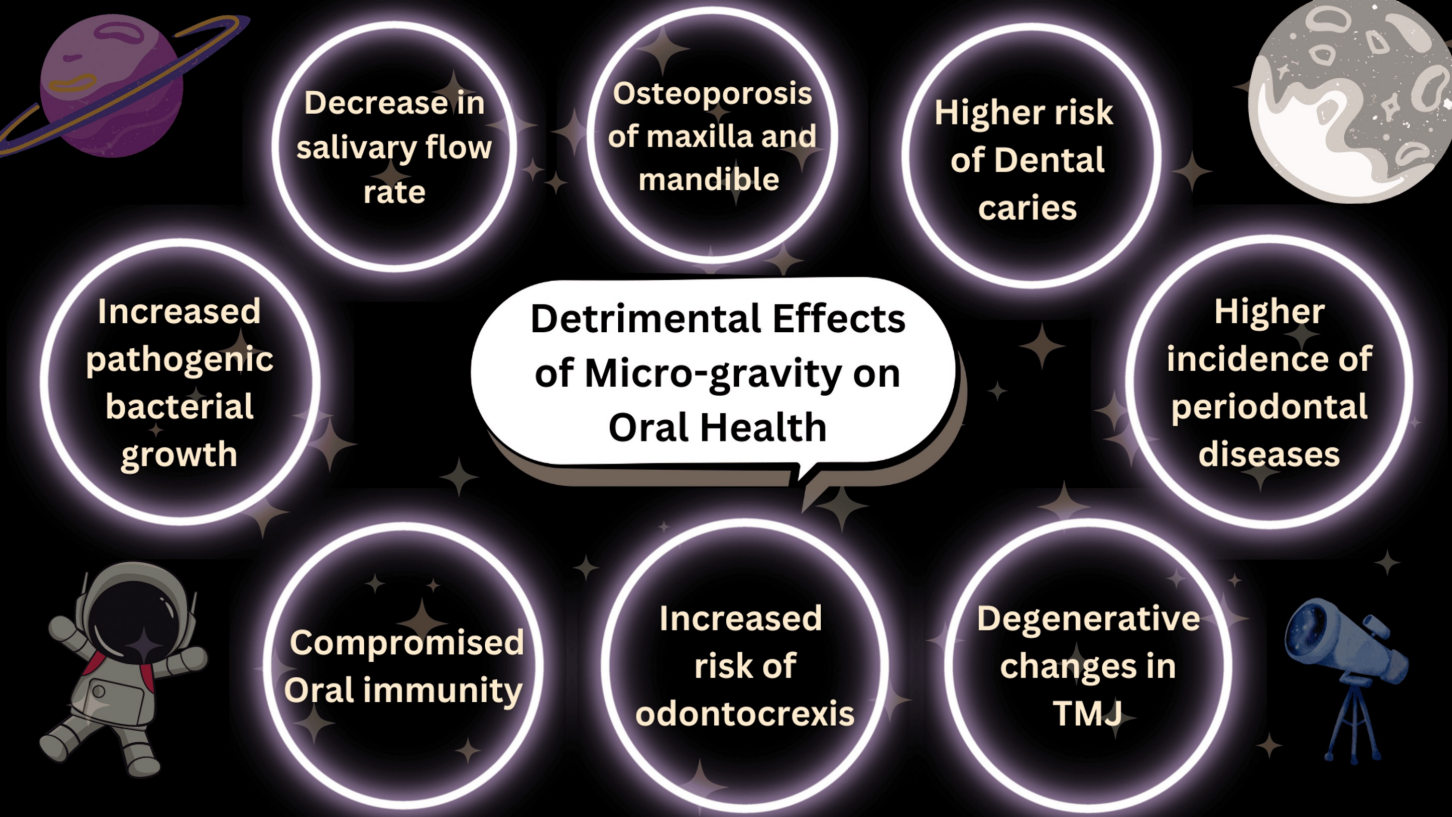
Detrimental effects of microgravity on oral health TMJ: temporomandibular joint

Figure 1 illustrates the adverse effects of microgravity on oral health.

Saliva

Microgravity alters the body's fluid distribution and balance, leading to decreased salivary flow, which can cause xerostomia (dry mouth). This condition is exacerbated by increased bone resorption, which raises the salivary saturation of calcium salts like calcium oxalate and calcium phosphate, heightening the risk of sialolithiasis. Environmental and dietary factors in space also influence salivary composition, increasing the likelihood of sialolithiasis [9].

The normal salivary flow rate is 0.4 ml/min and a flow rate of <0.1 ml/min is considered to be associated with some form of pathology, while during stimulation, <0.5 ml/min is considered pathological. Research by Rai et al. under simulated microgravity conditions demonstrated a significant reduction in unstimulated salivary flow rates over six weeks [10]. This decrease in salivary secretion is attributed to the reduced activity of the submandibular gland's muscles in microgravity [11]. The precipitation of calcium salts due to increased bone resorption further raises the risk of salivary stone formation in these conditions [12].

Oral Bacteria

Microgravity conditions influence oral bacteria dynamics significantly. The counts of Mycoplasma and cariogenic Streptococci increase, while those of enteric bacilli decrease. This environment promotes biofilm formation, enhancing bacterial resistance to antimicrobial agents. Escherichia coli and Staphylococcus aureus, for instance, exhibit increased antibiotic resistance due to thicker cell walls. The virulence of these microbes combined with a compromised immune system can lead to severe oral health issues such as periodontitis, delayed wound healing, and potentially cancerous lesions, a condition informally referred to as "space AIDS syndrome" [3].

Cheng et al. conducted a study on the effects of microgravity on Streptococcus mutans (S. mutans), comparing biofilms formed by S. mutans in microgravity (MSµg) and normal gravity (MSg). Using a superconducting magnet to simulate microgravity, the bacteria were incubated at 37 °C with cultures sampled hourly for 10 hours. The findings revealed that MSg exhibited enhanced acid resistance compared to MSg. Confocal laser scanning microscopy showed that the biofilm of MSµg was denser, though cell growth and acid production did not significantly differ from those grown under normal gravity [3].

In a study conducted during a Mars analog mission at the Mars Desert Research Station, USA, the bacterial count of Streptococcus mutans in dental plaque was measured in 12 healthy volunteers. The counts were recorded before and after two weeks. The results showed a significant increase in the S. mutans count (CFU/mg of plaque) after the mission compared to the baseline values [13].

Oral Immunity

During a simulated Mars mission involving 12 volunteers, researchers measured salivary immunoglobulins (IgA, IgM, and IgG) at one and two weeks. The study revealed that all measured immunoglobulins displayed lower levels compared to their baseline values. This indicates that oral immunity is compromised in microgravity environments, suggesting a diminished ability to fight oral pathogens under these conditions [13].

Odontocrexis

Odontocrexis refers to the phenomenon where pre-existing dental restorations, such as fillings or crowns, may fail when exposed to high-altitude environments. This is typically due to the unintentional expansion of gases trapped beneath the restorations. The increased pressure can lead to what is colloquially known as a "tooth explosion," where the restoration is forcefully dislodged from the tooth due to gas expansion [14-15].

When exposed to high altitude, pre-existing leaky restorations or recurrent caries lesions beneath the restoration might induce a tooth explosion due to the inadvertent expansion of gas trapped behind the restorations [16].

Periodontal Health

Microgravity and increased free radicals in space enhance pathogen virulence and reduce immunity, adversely affecting the periodontium and increasing the risk of periodontal diseases among astronauts. Studies have shown a 20% decrease in osteoblastic markers like alkaline phosphatase and osteocalcin and an increase in osteoclast activity markers such as cathepsin and metalloproteinases 8 and 9 after three months in altered gravity conditions. This imbalance in bone remodeling leads to decreased bone formation and increased resorption [17]. At high altitudes, reduced salivation contributes to a higher incidence of periodontal diseases. Factors such as poor oral hygiene, nervousness, and fatigue exacerbate these risks among crew personnel [18].

Dental Caries

The prevalence of dental caries increases significantly in microgravity due to various physiological changes experienced by astronauts. In the absence of gravity, there is a reduction in the amount of saliva, which normally protects dental tissues. This decrease is attributed to reduced activity in the submandibular muscles and alterations in saliva composition, specifically lower levels of protective antioxidants. Additionally, the altered salivary composition and a general weakening of the immune system enhance the virulence of pathogens, exposing enamel to a higher risk of cavitation [17]. Furthermore, studies indicate that caries, particularly in the mandibular anterior teeth, is higher in space compared to Earth due to decreased salivation [19].

Effect on Facial Bones

Under microgravity conditions, osteoporosis of both the maxilla and mandible has been observed. This bone demineralization results from an imbalance between bone formation and resorption processes. In microgravity, there is a noted decrease in Macrophage inflammatory protein-1 (MIP-1) alpha level, serving as a potential marker for bone loss. The rate of demineralization in such environments is about 1-2% of total bone mass per month. The loss of bone mass varies across different skeletal sites, depending on the bone and mechanical loading, leading to a reduced ability of bones to withstand a lack of gravity and an increased susceptibility to fractures [18].

Temporomandibular Joint

Morphological and histological studies of the temporomandibular joint (TMJ) indicate that it is not ideally suited to bear stress. When subjected to prolonged and increased stress, often resulting from certain parafunctional oral habits, there can be spasmatic hyperactivity in the mandibular musculature. This condition may lead to degenerative changes in the joint by exerting abnormal pressure on the fibrous tissue of the articular disc [12].

Dental Implants

A study focused on peri-implant bone changes in a French astronaut with a dental implant who spent six months on Russia's Mir space station. Measurements of the implant and peri-implant bone levels were taken by two examiners before and after the flight, and again after a recovery period. Periapical radiographs revealed that the peri-implant bone levels remained stable throughout the duration of microgravity, and the implant continued to function effectively without any complications [17].

Restorative Dentistry

Research has indicated that dental pain experienced during flight might be due to the expansion of air trapped under fillings, exerting pressure on the pulp via the dentinal tubules. Temperature changes in flight can also impact dental fillings. It was noted that thermal contraction in amalgam restorations due to low temperatures at high altitudes [20]. Additionally, inhalation of pure oxygen may corrode dental amalgam [21].

In space, direct and indirect pulp capping is discouraged due to constant barometric pressure changes, affecting any voids in the material under Boyle’s law [22]. On the International Space Station (ISS), Cavit is used as a temporary filling material, but its durability is limited to one month, making it unsuitable for longer missions [3].

Endodontic Treatment

Endodontic care in space must tackle unique challenges not present on Earth. Traditional treatments, such as direct pulp capping, are often unsuitable due to the effects of microgravity, which can cause complications like facial emphysema and apical extrusion [23]. In these conditions, maintaining the integrity of dental restorations, such as crowns, becomes critical but difficult due to the variability in pressure that affects the performance of luting cements.

Lyons and associates conducted a comparative study of zinc phosphate, glass ionomer, and resin cement under conditions simulating space. The findings revealed that resin cement provided superior performance in terms of retention and resistance to microleakage. This study indicated that zinc phosphate and glass ionomer cement, while common on Earth, were prone to failure under simulated space conditions due to their susceptibility to porosity changes with pressure fluctuations [22, 24]. 

The study further evaluated full-cast crowns attached to extracted teeth exposed to pressure cycling, simulating the changing pressures in space environments. The results confirmed that crowns fixed with zinc phosphate or glass ionomer cement exhibited increased microleakage and reduced retention post-pressure cycling, particularly those using zinc phosphate. In contrast, crowns using resin cement showed no microleakage and maintained their retention, underscoring the importance of selecting appropriate materials that can withstand the unique conditions of space [3].

Prosthodontic Considerations

In the field of prosthodontics, retention of dental appliances like dentures and crowns is crucial and is significantly influenced by factors such as atmospheric pressure, adhesion, and gravity. Specifically, when barometric pressure decreases, the retention of complete dentures is notably impaired, affecting their stability and function [24]. This issue is also pertinent in the retention of dental crowns, where changes in atmospheric pressure can negatively affect the microtubules within the cement layer, leading to decreased crown retention over time [23].

Research indicates that resin cement is preferable for securing crowns and fixed partial dentures, particularly in environments with fluctuating pressures, such as those encountered by divers. Furthermore, the shift towards implant-supported prostheses over removable ones has been significant, especially in contexts where maintaining consistent retention is challenging. Implant-supported prostheses offer superior retention and speech clarity, making them highly beneficial in environments with variable barometric pressures [25]. These advancements highlight the importance of choosing appropriate materials and techniques in prosthodontics to adapt to different environmental challenges.

Oral Surgical Considerations

In oral surgery, especially following the extraction of maxillary teeth, surgeons must assess for any oroantral communications. These communications can lead to complications such as sinusitis if the patient is exposed to environments with fluctuating pressures, such as those experienced in high-altitude or underwater settings [16]. Recent advancements in medical technology have brought about more effective methods for controlling hemorrhage. Studies, including one by Rhee et al., highlight the benefits of tissue sealant bandages, which are composed similarly to fibrin glue. These bandages have been shown to significantly reduce blood loss when compared to traditional gauze, offering a substantial improvement in managing surgical bleeding. More efficient bandages and dressings for hemorrhage control have been recently introduced [26]. These developments are pivotal for ensuring the safety and efficacy of oral surgical procedures in challenging environmental conditions.

Barodontalgia

Barodontalgia is a toothache caused by a variation in barometric pressure in an asymptomatic tooth. Pain is sharp or squeezing in nature. Pain occurring on both ascend and descend is related to the periapical disease. During flight, on ascending, it is associated with vital pulp tissue, and on descent, it is associated with pulp necrosis. The pathogenesis of barodontalgia was described by Strohaver in 1972, and he divided it into two main broad groups: direct and indirect types. In the direct barodontalgia, the reduced atmospheric pressure leads to a direct effect on a tooth on the affected tooth and is localized, moderate to severe, which usually develops during takeoff, whereas, in the indirect type, pain occurs due to the stimulation of the superior alveolar nerves at the time of maxillary barosinusitis and is dull, poorly defined, involving the posterior teeth, and develops during landing [27].

Air can enter the teeth by being forced in through carious lesions or defective restoration margins. During ascent, as the atmospheric pressure decreases, trapped gases may expand and enter dentinal tubules, thus stimulating the nociceptors in the pulp or even driving some of the pulp chamber contents out of the apex [3]. Rauch identified the following predisposing factors: acute or chronic periapical infection, caries, deep restorations, residual dental cysts, sinusitis, and a history of recent surgery [28].

Masticatory Muscle Activity

The reduction in the use of antigravity muscles in a weightless environment causes muscles to weaken and deteriorate. The masseter, temporalis, and medial pterygoid are the mandible-elevating muscles that function against gravitational forces and hence are more susceptible to atrophy. Phillippou et al. compared the responses of masticatory and appendicular muscles to microgravity using mice aboard the Space Shuttle Space Transportation System-135. Results suggested that the tibialis anterior muscle showed a decreased mass, decreased phosphorylated (P)-Akt, and increased atrogene expression after 13 days of space flight. In contrast, Masseter exhibited no notable alterations in mass, but there is an increase in P-focal adhesion kinase, P-Akt, and expression of atrogene. This suggested that distinct muscles showed different set points of adaptation, with the masticatory muscle being more stable than the tibialis anterior [29].

Solutions for dental considerations in space

Space exploration presents unique and formidable challenges to maintaining oral health, exacerbated by the microgravity environment, radiation exposure, limited access to traditional dental care, and altered physiological conditions. Despite these challenges, significant advancements and interdisciplinary collaborations offer promising solutions to ensure the dental well-being of astronauts during extended missions.

Microgravity affects various aspects of dental health, from saliva production to immune system responses and bone density. Reduced salivary flow and changes in its composition increase the risk of dental caries and periodontal disease. The enhanced virulence of oral bacteria and compromised immune function further exacerbate these risks, leading to conditions like periodontitis and potentially severe oral infections. Additionally, the structural integrity of bones, including the maxilla and mandible, is compromised due to decreased osteoblastic activity and increased bone resorption, raising concerns about osteoporosis and the overall stability of dental structures [30].

Addressing these challenges requires a multifaceted approach. Innovations in dental materials and technologies, such as resin-based cement, 3D-printed dental prostheses, and advanced tissue sealant bandages, provide effective solutions for maintaining dental restorations and managing hemorrhages in space. The development and use of specialized tools, like ergonomic toothbrushes, help maintain oral hygiene despite the absence of gravity. These tools are designed to function efficiently in a microgravity environment, ensuring that astronauts can continue their oral care routines effectively [30].

Preventive measures and training programs are crucial components of maintaining oral health during space missions. Astronauts undergo comprehensive training in oral hygiene practices tailored to the unique conditions of space. This includes the use of antimicrobial mouth rinses, the adaptation of diets to minimize cariogenic risks, and regular monitoring of oral health. Furthermore, emergency dental kits equipped with temporary filling materials, pain relief medications, and instructional guides are essential for addressing unexpected dental issues that may arise during a mission [30].

The Inflight Medical Support System (IMSS) for Skylab was designed to provide the on-board physician or Scientist Pilot with medical equipment adequate to make a diagnostic assessment of those injuries or illnesses most likely to occur in the Skylab environment. The Inflight Medical Support System contained equipment and medical kits with over 1300 different items, and for the first time, a dental kit. It also included a manual with line drawings of complete intraoral radiographs of each crewmember as well as integrated, illustrated diagnostic and treatment procedures [8].

Future perspectives

Interdisciplinary collaborations among dental professionals, biomedical engineers, and space agencies are vital for advancing space dentistry. These collaborations focus on the research and development of new materials, techniques, and protocols tailored to the space environment. For example, the study of osteoblast and osteoclast behavior in microgravity provides insights into bone remodeling processes and informs the development of countermeasures to prevent bone loss. Similarly, understanding the impact of microgravity on saliva production and composition leads to better strategies for managing dry mouth and reducing the risk of dental diseases.

As humanity continues to push the boundaries of space exploration, the health and well-being of astronauts remain a top priority. The implementation of comprehensive dental care protocols, combined with ongoing research and technological advancements, ensures that astronauts are well-equipped to maintain their oral health during long-duration missions. These efforts not only safeguard the health of space travelers but also contribute to the broader field of dental science, providing insights and innovations that can benefit dental care on Earth.

## Conclusions

While space exploration poses significant challenges to oral hygiene and dental care, the integration of innovative solutions and interdisciplinary efforts offers a promising pathway to maintaining astronauts' dental health. By understanding and addressing the unique conditions of space, humanity can continue to explore the cosmos while ensuring the well-being of those who venture beyond our planet. This commitment to health and innovation is essential for the success of future space missions and the advancement of dental science.
